# A Retrospective Observation of Treatment Outcomes Using Decitabine-Combined Standard Conditioning Regimens Before Transplantation in Patients With Relapsed or Refractory Acute Myeloid Leukemia

**DOI:** 10.3389/fonc.2021.702239

**Published:** 2021-08-24

**Authors:** Yuhang Li, Longcan Cheng, Chen Xu, Jianlin Chen, Jiangwei Hu, Na Liu, Sanchun Lan, Jing Xie, Ting Sun, Lei Wang, Yu Zhang, Yao Sun, Shuiping Chen, Liangding Hu

**Affiliations:** ^1^Department of Hematology, 5th Medical Center of Chinese PLA General Hospital, Beijing, China; ^2^Department of Hematology, Hainan Hospital of Chinese PLA General Hospital, Sanya, China; ^3^Department of Laboratory Medicine, 5th Medical Center of Chinese PLA General Hospital, Beijing, China

**Keywords:** hypomethylating agents, decitabine, allogeneic hematopoietic stem cell transplantation, conditioning regimen, relapsed/refractory acute myeloid leukemia

## Abstract

Hypomethylating agents, decitabine (DAC) and azacitidine, can act as prophylactic and pre-emptive approaches after allogeneic hematopoietic stem cell transplantation (allo-HSCT) and a non-intensive bridging approach before allo-HSCT. However, they are rarely used as a part of conditioning regimens in patients with relapsed or refractory acute myeloid leukemia (AML). This retrospectively study included a total of 65 patients (median, 37; range, 13–63) with relapsed or refractory AML who were treated by allo-HSCT after myeloablative conditioning regimens without or with DAC (high-dose DAC schedule, 75 mg/m^2^ on day −9 and 50 mg/m^2^ on day −8; low-dose DAC schedule, 25 mg/m^2^/day on day −10 to −8). DAC exerted no impact on hematopoietic reconstitution. However, patients who were treated with the high-dose DAC schedule had significantly higher incidence of overall survival (OS, 50.0%) and leukemia-free survival (LFS, 35.0%), and lower incidence of relapse (41.1%) and grade II–IV acute graft versus host disease (aGVHD, 10.0%) at 3 years, when compared with those treated with standard conditioning regimens or with the low-dose DAC schedule. In conclusion, high-dose DAC combined with standard conditioning regimens before allo-HSCT is feasible and efficient and might improve outcomes of patients with relapsed or refractory AML, which provides a potential approach to treat these patients.

## Introduction

With significant advances in the treatment of acute myeloid leukemia (AML), 60%–80% of AML patients can achieve complete remission (CR) after standard induction chemotherapy consisting of anthracyclines and cytarabine (1). However, only 20%–30% of the patients with CR can achieve long-term leukemia-free survival (2), and over 50% of patients with CR will suffer from disease relapse, even death within 1 year (3). For these patients, allogeneic hematopoietic stem cell transplantation (allo-HSCT) provides the only curative option, by using an appropriate conditioning regimen that is critical to influence the success of allo-HSCT (4). The general goals of conditioning regimens are to clear residual leukemia cells, suppress the immune system, and successfully engraft donor stem cells (5). Due to high relapse rates and transplant-related mortality (TRM) in relapsed or refractory AML patients, traditional conditioning regimens consisting of cyclophosphamide (CY) with busulfan (BU) or total body irradiation (TBI) remain far from satisfactory.

Theoretically, minimizing the disease load before allo-HSCT is the most effective way to decrease the relapse rate. Hypomethylating agents (HMA), decitabine (DAC) and azacitidine, are DNA methyltransferase inhibitors that can inhibit DNA methyltransferase, reverse DNA methylation, and reactivate tumor-suppressor genes in leukemia cells (6). Meanwhile, DAC can increase the expression of class I human leukocyte antigens (HLA), HLA-DR, and CD80 (7, 8), which might increase the susceptibility of malignant cells to the graft versus leukemia effect (9). DAC can also alleviate the graft versus host disease (GVHD) by modulating regulatory T cells (10). These advantages of DAC make it an attractive drug in conditioning regimens before allo-HSCT.

Previous studies have investigated the feasibility and effectiveness of using HMA as a bridging therapy prior to transplantation in patients with myelodysplastic syndrome (MDS) and/or AML (11). In this retrospective study, we, for the first time, investigated the efficacy and dose schedules of HMA as part of standard conditioning regimens before allo-HSCT in patients with relapsed or refractory AML. Because azacitidine, which was approved by China Food and Drug Administration (FDA) in 2017, was not available during this study, DAC (approved in 2008 in China) was used here.

## Materials and Methods

### Patients

Sixty-five patients with relapsed or refractory AML from April 2006 to May 2016 were included in this retrospective study. All patients were diagnosed according to the 2008 revision of the World Health Organization (WHO) classification of myeloid neoplasms and acute leukemia (12). Patients who failed to achieve CR after one or two cycles of induction chemotherapy were considered as refractory AML. Relapsed AML was defined as follows: (i) bone marrow (BM) blasts ≥5% after remission, (ii) blasts were found in the peripheral blood again, and (iii) extramedullary relapse. Sixty-five consecutive patients were grouped based on conditioning regimens before allo-HSCT. Twenty-seven patients before January 2013 (between April 2006 and December 2012) received standard conditioning regimens (SCs) consisting of busulfan (BU) plus cyclophosphamide (CY) or CY plus total body irradiation (TBI) and served as the control group. Thirty-eight patients since January 2013 (between January 2013 and May 2016) received DAC-involved regimens, 20 of whom in one ward received high-dose DAC combined with standard conditioning regimens (HDD-SC) and 18 of whom in the other ward received low-dose DAC combined with standard conditioning regimens (LDD-SC). The patient details are described in **Table 1**.

**Table 1:** Demographics of patients in the HDD-SC group, the LDD-SC group, and the SC group.

Variables	HDD-SC (n = 20)	LDD-SC (n = 18)	SC (n = 27)	*p*-values
Age, median (range)	42 (24–57)	35 (14–63)	36 (13–57)	0.512
Gender				0.261
Male, n (%)	16 (80.0%)	10 (55.6%)	19 (70.4%)	
Female, n (%)	4 (20.0%)	8 (44.4%)	8 (29.6%)	
AML type				0.660
Primary AML, n (%)	11 (55.0%)	11 (61.1%)	19 (70.4%)	
Therapy-related AML, n (%)	9 (45.0%)	7 (38.9%)	8 (29.6%)	
AML classification				0.718
Refractory AML, n (%)	11 (55.0%)	11 (61.1%)	18 (66.7%)	
Relapsed AML, n (%)	9 (45.0%)	7 (38.9%)	9 (33.3%)	
BM blast percentage				0.472
<20%, n (%)	11 (55.0%)	8 (44.4%)	10 (37.0%)	
≥20%, n (%)	9 (45.0%)	10 (55.6%)	17 (63.0%)	
Time from diagnosis to transplant (month), median (range)	5.75 (1.5–35)	6.5 (2–24)	9 (3–124)	0.430
HLA type				0.147
Matched related, n (%)	10 (50.0%)	5 (27.8%)	14 (51.9%)	
Mismatched related, n (%)	5 (25.0%)	2 (11.1%)	1 (3.7%)	
Matched unrelated, n (%)	3 (15.0%)	4 (22.2%)	4 (14.8%)	
Mismatched unrelated, n (%)	2 (10.0%)	7 (38.9%)	8 (29.6%)	
Gender relationship				0.061
Matched, n (%)	11 (55.0%)	6 (33.3%)	14 (51.9%)	
Unmatched, n (%)	9 (45.0%)	9 (50.0%)	13 (48.1%)	
Unknown, n (%)	0 (0%)	3 (16.7%)	0 (0%)	
ABO blood type				0.300
Matched, n (%)	8 (40.0%)	6 (33.3%)	15 (55.6%)	
Mismatched, n (%)	12 (60.0%)	12 (66.7%)	12 (44.4%)	
Conditioning regimen				0.818
CY/TBI, n (%)	15 (75.0%)	15 (83.3%)	21 (77.8%)	
BU/CY, n (%)	5 (25.0%)	3 (16.7%)	6 (22.2%)	
Cytogenetics for AML				0.635
High, n (%)	5 (25.0%)	1 (5.6%)	5 (18.5%)	
Intermediate, n (%)	8 (40.0%)	11 (61.1%)	15 (55.6%)	
Low, n (%)	1 (5.0%)	2 (11.1%)	3 (11.1%)	
Unknown, n (%)	6 (30.0%)	4 (22.2%)	4 (14.8%)	
Disease status at transplantation				0.823
CR, n (%)	2 (10.0%)	3 (16.7%)	4 (14.8%)	
NR, n (%)	18 (90.0%)	15 (83.3%)	23 (85.2%)	
Infused cells, median (range)				
CD34+ (×10^6^/kg)	4.63 (1.69–11.00)	4.86 (0.85–9.71)	4.44 (1.32–12.10)	0.763
CD3+ (×10^8^/kg)	3.67 (1.40–7.83)	2.47 (0.75–6.25)	3.04 (1.01–6.50)	0.222

This study was approved by the ethical committee of the 5th Medical Center of Chinese PLA General Hospital (No. KY-2019-12-57). Informed consents were obtained from all of the patients or from a parent or guardian of the patients under 16 years old, in accordance with the Declaration of Helsinki.

### Conditioning Regimens

All patients received the myeloablative conditioning regimens. Patients in the SC group received BU plus CY or CY plus TBI. BU/CY was administered as follows: BU 0.8 mg/kg q6h intravenously on day −7 to −4, CY 60 mg/kg/day on day −3 to −2. CY/TBI was administered as follows: CY 60 mg/kg/day on day −4 to −3, TBI 5.0 Gy/day on day −2 to −1. Two different DAC doses were determined based on previous studies (13–15). Patients in the HDD-SC group received the standard preparative regimen (as described above) plus high-dose DAC (75 mg/m^2^ on day −9 and 50 mg/m^2^ on day −8 intravenously for 3 h). Patients in the LDD-SC group received the standard preparative regimen (as described above) plus low-dose DAC (25 mg/m^2^/day on day −10 to −8 intravenously for 3 h).

### Graft *Versus* Host Disease Prophylaxis and Management

Patients receiving HLA-matched sibling grafts were given cyclosporin A (CSA, 1.5 mg/kg/day intravenously until hematopoietic reconstitution, then administered orally to maintain the blood concentration between 150 and 200 ng/ml) combined with a short course of methotrexate (MTX, 15 mg/m^2^ on day +1, 10 mg/m^2^ on day +3 and day +6). Patients receiving HLA-mismatched sibling grafts were given antithymocyte globulin (ATG, 2.5 mg/kg/day on day −4 to −2) combined with CSA and a short course of MTX. Patients receiving unrelated grafts were given a regimen consisting of CSA, a short course of MTX, MMF (1.5 g/m^2^/day on days 0–28), and CD25 antibody (basiliximab, 20 mg/day on days 0, 4, and 8).

### Infection Prevention and Supportive Care

All patients were cared for in sterile laminar flow wards. Ganciclovir and trimethoprim sulfamethoxazole were routinely administered to prevent viruses and pneumocysis carinii infection, respectively. Patients without invasive fungal infections (IFIs) were administered with fluconazole to prevent fungal infections, while those with IFI were administered with primary effective antifungal drugs. Patients with platelet counts <20 × 10^9^/L were transfused with leukodepleted and irradiated platelets, and those with hemoglobin <70 g/L were transfused with leukodepleted and irradiated red blood cells. Liver veno-occlusive disease (VOD) was prevented by a combination of alprostadil, ursofalk, and heparin.

### Evaluations and Definitions

Leukemia*-*free survival (LFS) was defined as the time from transplantation to the date of relapse or death of any cause. Overall survival (OS) was defined as the time from transplantation to death of any cause or last follow-up. Relapse was defined as BM blasts ≥5%, appearance of leukemia cells in peripheral blood, or extramedullary leukemia cell infiltration after CR. Acute GVHD (aGVHD) was diagnosed and graded according to the criteria of Przepiorka et al. (16). Chronic GVHD (cGVHD) were classified according to the National Institutes of Health scoring system (17). Neutrophil recovery was defined as the first of three consecutive days with an absolute neutrophil count >0.5 × 10^9^/L. Platelet recovery was defined as the first of seven consecutive days with an untransfused platelet count >20 × 10^9^/L.

### Statistical Analysis

Cumulative incidence curves of OS, LFS, relapse, and grade II–IV aGVHD were plotted using the Kaplan–Meier method, and the differences in OS, LFS, relapse, and grade II–IV aGVHD between groups were calculated with the log-rank test. Data with a normal distribution were presented as mean standard deviation (SD), and medians were used to describe data with non-normal distribution. Multivariate analysis was performed to calculate hazard ratio (HR) and 95% confidence interval (CI) by using Cox proportional hazard regression models. For all analysis, p < 0.05 in two-tailed test was considered as statistically significant. All statistical analyses were performed with SPSS 20.0.

## Results

### Characteristics of Patients, Diseases, and Transplant

The median ages of patients in the SC group, the HDD-SC group, and the LDD-SC group were 36 (range, 13–57), 42 (range, 24–57), and 35 (range, 14–63), respectively. The median follow-up times after transplantation were 9 months (range, 1–137), 40 months (range, 1–86), and 5 months (range, 1–93), respectively. Patient characteristics are summarized in **Table 1**. There were no significant differences in variables of age, gender, AML type, AML classification, BM blast percentage, time from diagnosis of relapsed or refractory AML to transplant, donor HLA type, gender relationship, ABO blood type, conditioning regimen, cytogenetics, disease status at transplantation, and infused cells between any two groups (p > 0.05).

### Neutrophil and Platelet Engraftment

**Figure 1:**
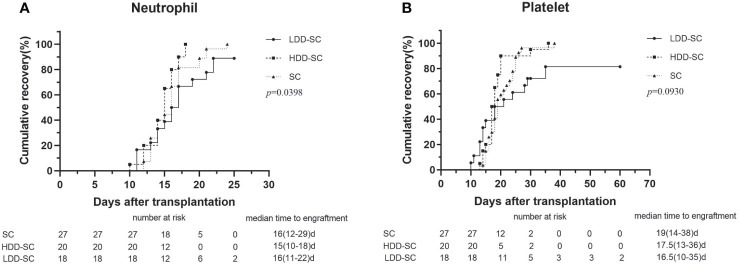
Cumulative incidence of engraftment. **(A)** Time to neutrophils recovery above 0.5 × 10^9^/L. **(B)** Time to platelets recovery above 20 × 10^9^/L.

The median times to neutrophil engraftment in the SC group, the HDD-SC group, and the LDD-SC group were 16 days (range, 12–29), 15 days (range, 10–18), and 16 days (range, 11–22) (**Figure 1A**), respectively, which showed no significant differences among the three groups (p > 0.05). The median times to platelet engraftment in the SC group, the HDD-SC group, and the LDD-SC group were 19 days (range, 14–38), 17.5 days (range, 13–36), and 16.5 days (range, 10–35) (**Figure 1B**), respectively, which also showed no significant differences among the three groups (p > 0.05). Notably, two patients failed to achieve neutrophil and platelet engraftment, and another two patients achieved neutrophil engraftment but failed to achieve platelet engraftment in the LDD-SC group.

### Remission Rates, OS, LFS, and Relapse

**Figure 2:**
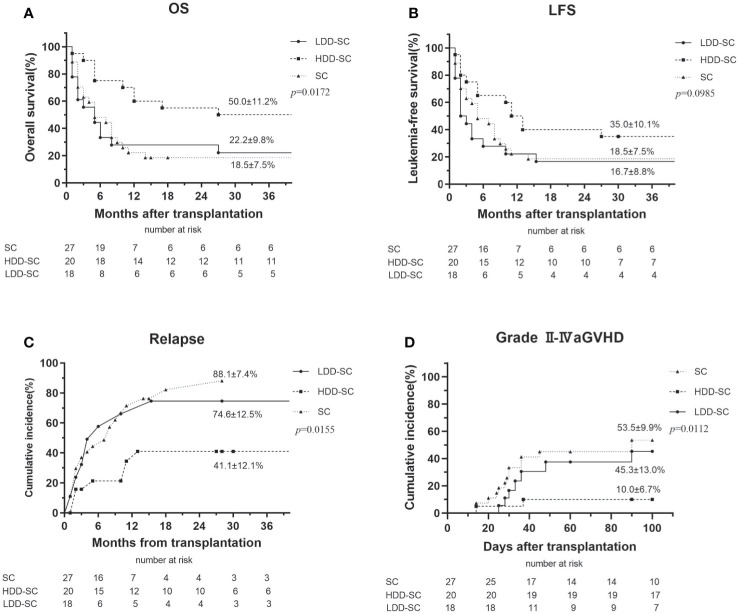
Clinical outcomes for all patients. **(A)** Overall survival (OS), **(B)** leukemia-free survival (LFS), **(C)** relapse, and **(D)** grade II–IV acute graft *versus* host disease (aGVHD).

CR at 3 months after allo-HSCT was 75.0%, 57.1%, and 63.0% in the HDD-SC group, the LDD-SC group, and the SC group, respectively. The cumulative incidence of OS at 3 years in the HDD-SC group (50.0%) was significantly higher than that in the LDD-SC group (22.2%) or the SC group (18.5%) (p < 0.05), while there was no significant difference between the LDD-SC group and the SC group (p > 0.05) (**Figure 2A**). Similarly, the cumulative incidence of LFS at 3 years in the HDD-SC group (35.0%) was significantly higher than that in the LDD-SC group (16.7%) (p < 0.05), and no significant difference between the LDD-SC group and the SC group was observed (p>0.05) (**Figure 2B**). As for relapse, the cumulative incidence at 3 years in the HDD-SC group (41.1%) was significantly lower than that in the SC group (88.1%) or the LDD-SC group (74.6%) (p < 0.05), and no significant difference between the LDD-SC group and the SC group was observed (p > 0.05) (**Figure 2C**). However, the median time to relapse in the HDD-SC group was 11.0 months (range, 2–31), which was significantly longer than that in the LDD-SC group (median, 3.5 months; range, 1–15) or the SC group (median, 6.5 months; range, 1–15) (*p* < 0.05).

Similar results were observed when incidences of OS, LFS, and relapse at 1 year among the three groups were compared (**Table 2**).

**Table 2:** Comparison of OS, LFS, and relapse at 1 and 3 years among the HDD-SC group, the LDD-SC group, and the SC group.

Outcomes	Groups			*p-*value		
	LDD-SC	HDD-SC	SC	HDD-SC *vs.* LDD-SC	HDD-SC *vs.* SC	LDD-SC *vs.* SC
1-year OS	27.8 ± 10.6%	65 ± 10.7%	22 ± 8%	**0.0095**	**0.0045**	0.6106
3-year OS	22.2 ± 9.8%	50 ± 11.2%	18.5 ± 7.5%	**0.0213**	**0.0102**	0.6111
1-year LFS	22.2 ± 9.8%	50 ± 11.2%	22.2 ± 8%	**0.0409**	0.0651	0.4886
3-year LFS	16.7 ± 8.8%	35 ± 10.1%	18.5 ± 7.5%	**0.0478**	0.0903	0.4675
1-year relapse	66.1 ± 13.4%	34.5 ± 11.6%	68.7 ± 9.9%	0.0813	**0.0054**	0.5488
3-year relapse	74.6 ± 12.5%	41.1 ± 12.1%	88.1 ± 7.4%	**0.0414**	**0.0033**	0.6178

p values less than 0.05 were bold.

As for causes of death, 81.5% (22/27) of patient in the SC group died of leukemia relapse (n = 21) and sudden cardiac death (n = 1); 50.0% (10/20) of patients in the HDD-SC group died of severe infections (n = 4; 2 lung infection patients, 1 influenza A infection patient, and 1 bacteremia patient), leukemia relapse (n = 4), respiratory failure (n = 1), and sudden cardiac death (n = 1); and 77.8% (14/18) of patients in the LDD-SC group died of leukemia relapse (n = 9), severe infections (n = 4; 3 lung infection patients and 1 bacteremia patient), and severe aGVHD (n = 1).

### Graft *Versus* Host Disease

Sixty-three percent (17/27) of patients in the SC group developed aGVHD, 14 of whom were grades II–IV and 6 of whom were grades III–IV. Forty percent (8/20) of patients in the HDD-SC group developed aGVHD, two of whom were grades II–IV and none were grades III–IV. Of the patients in the LDD-SC group, 55.6% (10/18) developed aGVHD, seven of whom were grades II–IV and two of whom were grades III–IV. The incidence of grades II–IV aGVHD in the HDD-SC group (10.0%) was significantly lower than that in the LDD-SC group (45.3%) or the SC group (53.5%) (*p* < 0.05) (**Figure 2D**).

As for cGVHD, 37.0% (10/27), 5.0% (1/20), and 44.4% (8/18) of patients were observed in the SC group, the HDD-SC group, and the LDD-SC group, respectively.

### Multivariate Analysis

In a multivariate analysis of patients in the SC group and the HDD-SC group (**Table 3**), high dose of DAC was associated with lower relapse; BM blast (<20%) and high dose of DAC were associated with longer OS; BM blast (<20%) was associated with longer LFS; and high dose of DAC was associated with less grade II–IV aGVHD. Similar results were observed between the HDD-SC group and the LDD-SC group.

**Table 3:** Multivariate analysis of relapse, OS, LFS, and grade II–IV aGVHD between the HDD-SC group and the SC group.

Variables	Relapse			OS			LFS			Grade II–IV aGVHD		
	HR	95% CI	*p*-value	HR	95% CI	*p* -value	HR	95% CI	*p*-value	HR	95% CI	*p* -value
Recipient sex												
Female (reference)	1	–		1	–		1	–		1	–	
Male	0.733	0.234–2.298	0.595	0.722	0.264–1.97	0.524	0.696	0.265–1.828	0.461	3.523	0.751–16.532	0.110
Recipient age (years)												
≤35 (reference)	1	–		1	–		1	–		1	–	
>35	1.633	0.551–4.837	0.376	1.146	0.400–3.286	0.799	1.787	0.656–4.864	0.256	0.732	0.200–2.678	0.637
AML type												
Primary AML (reference)	1	–		1	–		1	–		1	–	
t-AML	1.352	0.474–3.852	0.573	1.194	0.457–3.118	0.717	1.284	0.516–3.197	0.590	1.550	0.460–5.228	0.480
AML classification												
Refractory (reference)	1	–		1	–		1	–		1	–	
Relapse	2.982	0.969–9.172	0.057	1.580	0.559–4.462	0.388	2.696	0.943–7.701	0.064	0.622	0.134–2.894	0.545
Disease status at transplantation												
NR (reference)	1	–		1	–		1	–		1	–	
CR	1.368	0.334–5.603	0.663	2.403	0.647–8.931	0.191	1.483	0.432–5.093	0.531	0.936	0.084–10.401	0.957
BM blast percentage												
<20% (reference)	1	–		1			1	–		1	–	
≥20%	2.285	0.805–6.491	0.121	3.623	1.240–10.585	**0.019**	2.621	1.022–6.720	**0.045**	3.184	0.732–13.853	0.123
Gender relationship												
Matched (reference)	1	–		1	–		1	–		1	–	
Mismatched	0.476	0.137–1.658	0.244	1.110	0.357–3.452	0.857	0.759	0.273–2.107	0.596	1.722	0.343–8.640	0.509
HLA type												
Matched (reference)	1	–		1	–		1	–		1	–	
Mismatched	0.902	0.271–3.007	0.867	0.254	0.062–1.036	0.056	0.582	0.184–1.842	0.357	0.902	0.143–5.704	0.913
ABO blood type												
Matched (reference)	1	–		1			1	–		1	–	
Mismatched	1.975	0.669–5.833	0.218	2.123	0.763–5.904	0.149	1.789	0.706–4.532	0.220	1.421	0.350–5.773	0.623
Conditioning regimens												
SC (reference)	1	–		1	–		1	–		1	–	
HDD-SC	0.342	0.121–0.963	**0.042**	0.331	0.128–0.857	**0.023**	0.580	0.243–1.386	0.220	0.114	0.020–0.659	**0.015**

p values less than 0.05 were bold.

## Discussion

AML presents in all ages with a slow rise in young adulthood (18). Although allo-HSCT provides a potentially curative option for AML, relapse remains as a main cause of treatment failure (19). Until recently, sequential therapeutic options consisted of palliative care, chemotherapy, cellular therapy, and second transplantation. HMA can act as a prophylactic and pre-emptive approach to avoid hematological relapse after allo-HSCT, and a non-intensive bridging approach before allo-HSCT (11, 20). The prophylactic approach aims to directly eliminate residual malignant cells and control disease activity until the donor immune system is sufficiently reconstituted to mediate the desired graft versus leukemia effect. The pre-emptive approach is initiated as soon as there is any evidence of relapse at a submicroscopical level to avoid conversion to frank hematological relapse. Despite the considerable progress of these new therapies, the treatment of patients with relapsed or refractory AML still remains as the most challenging obstacle (21). Particularly for those with relapsed or refractory AML in no remission (NR) status, any further salvage therapy to achieve CR might increase the risk of infections and organ toxicities (22). Previous studies have shown that DAC has antileukemia activity, negligible non-hematopoietic toxicity, and good tolerance (23–25). However, previous studies assessing HMA therapies before allo-HSCT, as a bridge to transplantation, mainly focus on elderly patients with AML or MDS (11). Compared with the few studies using DAC as a part of standard conditioning regimens before allo-HSCT (14, 15), the novelties in this study included more patients, AML patients only, and better clinical benefits. To the best of our knowledge, this is the first study to evaluate the outcomes of DAC combined with standard conditioning regimens before allo-HSCT in patients with relapsed or refractory AML.

DAC is confirmed to be well-tolerated in patients with relapsed or refractory AML, even in the cases with increased age and comorbid burden (26, 27). Considering that the maximum tolerated dose of DAC can reach up to 1,500–2,500 mg/m^2^ and no clear correlation between dosage and response has been found (13), a high-dose schedule (75 mg/m^2^ × 1 and 50 mg/m^2^ × 1) and a low-dose schedule (25 mg/m^2^/day × 3) of DAC, as part of standard conditioning regimens, were compared in allo-HSCT patients with relapsed or refractory AML in this study. We found that the two dose schedules had no impact on hematopoietic reconstitution when compared with the standard conditioning regimens (BU/CY and CY/TBI). In addition, the high-dose DAC schedule, but not the low-dose DAC schedule, provided significantly longer OS and LFS and significantly lower relapse and grade II–IV aGVHD. Multivariate analyses also suggested that high-dose DAC was associated with longer OS, lower relapse, and lower grade II–IV aGVHD. Compared to the FLAMSA-RIC regimens [fludarabine, amsacrine, and cytarabine (FLAMSA) followed by reduced-intensity conditioning (RIC)] in HSCT patients with relapsed or refractory AML or MDS, the high-dose DAC schedule in this study showed a higher 3-year OS and 3-year LFS (50.0% *vs*. 27.8%, 35.0% *vs*. 23.7%), although the 3-year OS and 3-year LFS in the SC group were lower (18.5% *vs*. 27.8%, 18.5% *vs*. 23.7%) (28), which suggested that high-dose DAC plus standard conditioning regimens might provide better outcomes than the FLAMSA-RIC regimens. The significant differences in clinical outcomes in the two DAC-involved groups might be due to dose-dependent apoptosis in myeloid leukemia cells and synergistic cytotoxicity or chemosensitization (29, 30), especially dose-dependent upregulation of PD-L1, PD-L2, PD-1, and CTLA4 expression (31).

Relapse remains as a main cause of treatment failure. The longer OS in the HDD-SC group in this study might be due to the longer median time to relapse (11 months), which is consistent with a previous report that patients who relapse <6 months after allo-HSCT have higher risk of mortality (32). Thus, early prophylactic therapy is crucial to decrease or delay relapse for patients with relapsed or refractory AML so as to avoid worse survival. DAC can augment NK reactivity by activating the killer Ig-like receptor (KIR) on NK cells (33), therefore is associated with a low rate of leukemia relapse in allo-HSCT patients (34, 35). Meanwhile, proper, but not excessive, NK cells exert greater clinical benefits in a DAC-involved conditioning therapy (36). The improved clinical benefits of the high-dose DAC schedule in this study might be highly correlated with synergistic effects of DAC and chemotherapy agents in killing leukemic cells. On the other hand, HMA can activate the expression of cancer-testis antigens (CTAs) and human endogenous retroviruses (ERVs) suppressed by DNA methylation, leading to production of neoantigens in treated cells, which facilitate targeting by the host immune system. Activation of ERV can also lead to a state of viral mimicry and induce the innate immune response (37). We did not observe more frequent severe aGVHD in this study, which suggested that DAC might selectively increase the expression of tumor-associated antigens or CTA on leukemic cells without increasing the corresponding expression in healthy tissues (e.g., intestine, skin, liver cells). Although high-dose DAC might cause high degree of hematological toxicity with prolonged myelosuppression (38), it is worth investigating the dose schedules and mechanism of DAC to further improve its clinical benefits as part of conditioning regimens before allo-HSCT in patients with relapsed or refractory AML.

As for causes of death in this study, infection was the leading one in the HDD-SC group and the LDD-SC group (40.0%, 4/10; 28.6%, 4/14), which was consistent with a previous study (33.3%, 5/15) conducted in the similar cohort using DAC plus myeloablative allo-HSCT (39). The high infection rates might be due to the high DAC schedules, according to a previous study comparing the 10-day DAC exposure with the 5-day DAC exposure in older AML patients (40). Thus, optimization of DAC schedules, and infection screening and antibiotic prophylaxis, could be helpful to reduce the infection risk during the DAC-involved myeloablative allo-HSCT.

There are limitations in this study. First, the results presented here should be interpreted with caution due to the retrospective characteristic of the study and the limited number of patients in each group. Second, the imbalance and relatively short follow-up time of some patients might bias the results. Therefore, a prospective study, which includes multiple centers and more relapsed or refractory AML patients, will be conducted for confirmation of the promising observations. Dose schedules of DAC, as part of standard conditioning regimens before allo-HSCT, will be optimized to make a better balance between its side effects and antileukemia activities to further improve treatment outcomes. Nevertheless, our results show at least the feasibility and efficacy of DAC-involved standard conditioning regimens before allo-HSCT in patients with relapsed or refractory AML.

In summary, high-dose DAC combined with standard conditioning regimens before allo-HSCT is feasible and efficient and might improve outcomes (higher incidences of OS and LFS, lower incidences of relapse, and grade II–IV aGVHD) in patients with relapsed or refractory AML, which is suggested to be a potential approach to treat these patients.

## Data Availability Statement

The original contributions presented in the study are included in the article/supplementary material. Further inquiries can be directed to the corresponding authors.

## Ethics Statement

The studies involving human participants were reviewed and approved by the ethical committee of the 5th medical center of Chinese PLA general hospital. Written informed consent to participate in this study was provided by the participants’ legal guardian/next of kin. Written informed consent was obtained from the individual(s), and minor(s)’ legal guardian/next of kin, for the publication of any potentially identifiable images or data included in this article.

## Author Contributions

YL and LC designed and performed the study and collected and analyzed the data. CX, JC, JH, NL, SL, JX, TS, LW, YZ, and YS collected and analyzed the data. SC and LH designed the study and drafted and revised the manuscript. All authors contributed to the article and approved the submitted version.

## Funding

This study was supported by the Capital Characteristic Clinical Project of China (Z171100001017188).

## Conflict of Interest

The authors declare that the research was conducted in the absence of any commercial or financial relationships that could be construed as a potential conflict of interest.

## Publisher’s Note

All claims expressed in this article are solely those of the authors and do not necessarily represent those of their affiliated organizations, or those of the publisher, the editors and the reviewers. Any product that may be evaluated in this article, or claim that may be made by its manufacturer, is not guaranteed or endorsed by the publisher.
